# Enhancing α-secretase Processing for Alzheimer’s Disease—A View on SFRP1

**DOI:** 10.3390/brainsci10020122

**Published:** 2020-02-22

**Authors:** Bor Luen Tang

**Affiliations:** 1 Department of Biochemistry, Yong Loo Lin School of Medicine, National University of Singapore, Singapore 117596, Singapore; 2 NUS Graduate School for Integrative Sciences and Engineering, National University of Singapore, Singapore 119077, Singapore

**Keywords:** ADAM10, Alzheimer’s disease, amyloid β (Aβ), secreted-frizzled-related protein 1 (SFRP1)

## Abstract

Amyloid β (Aβ) peptides generated via sequential β- and γ-secretase processing of the amyloid precursor protein (APP) are major etiopathological agents of Alzheimer’s disease (AD). However, an initial APP cleavage by an α-secretase, such as the a disintegrin and metalloproteinase domain-containing protein ADAM10, precludes β-secretase cleavage and leads to APP processing that does not produce Aβ. The latter appears to underlie the disease symptom-attenuating effects of a multitude of experimental therapeutics in AD animal models. Recent work has indicated that an endogenous inhibitor of ADAM10, secreted-frizzled-related protein 1 (SFRP1), is elevated in human AD brains and associated with amyloid plaques in mouse AD models. Importantly, genetic or functional attenuation of SFRP1 lowered Aβ accumulation and improved AD-related histopathological and neurological traits. Given SFRP1′s well-known activity in attenuating Wnt signaling, which is also commonly impaired in AD, SFRP1 appears to be a promising therapeutic target for AD. This idea, however, needs to be addressed with care because of cancer enhancement potentials resulting from a systemic loss of SFRP1 activity, as well as an upregulation of ADAM10 activity. In this focused review, I shall discuss α-secretase-effected APP processing in AD with a focus on SFRP1, and explore the contrasting perspectives arising from the recent findings.

## 1. Introduction

**Figure 1:**
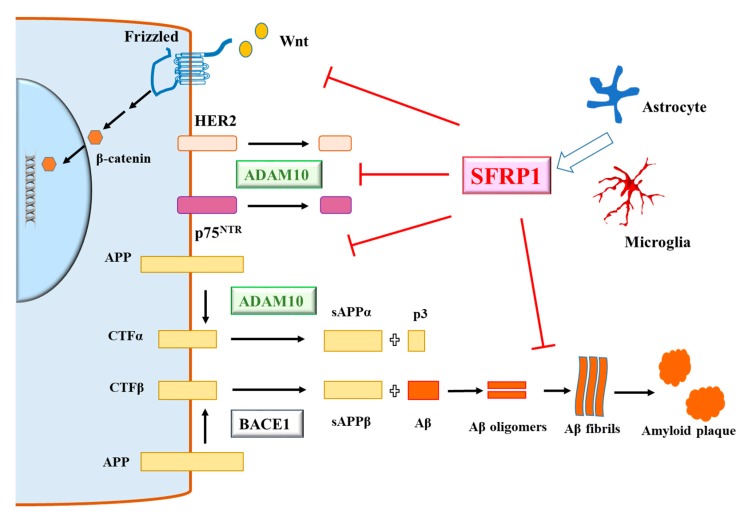
A schematic diagram illustrating the possible roles of secreted-frizzled-related protein 1 (SFRP1) in Alzheimer’s disease (AD). Astrocyte- and microglial (and other cell types)-secreted SFRP1 inhibit metalloproteinase domain-containing protein (ADAM10)-mediated non-amyloidogenic processing of amyloid precursor protein (APP). SFRP1 could also interfere with fibril/protofibril formation by amyloid β (Aβ), thus potentially preserving Aβ oligomers. SFRP1 also inhibits Wnt signaling, which is important for neuronal function and synaptic integrity. On the other hand, SFRP1 could be tumor suppressive via its inhibition of ADAM10’s sheddase activity, which liberates the ectodomain of growth/metastasis promoting proteins such as human epidermal growth factor receptor 2 (HER2) and the low affinity neurotrophin receptor (p75^NTR^). See text for more details.

Alzheimer’s disease (AD) [1] is the most prevalent form of age-associated dementia [2]. The amyloid cascade hypothesis [3], or a modified amyloid β (Aβ) oligomer hypothesis [4], postulated that AD etiology stems from Aβ peptides (particularly the toxic and aggregation-prone Aβ_1–42_) [5] that are produced by two sequential amyloidogenic proteolytic cleavages of the amyloid precursor protein (APP), first by the β-site APP-cleaving enzyme 1 (BACE1) [6] followed by γ-secretase [7]. The validity of the amyloid cascade hypothesis has been questioned, as it may be an oversimplification if Aβ’s pathological interaction with tau [8] is not taken into account, and because many amyloid-targeting therapeutic attempts have thus far proven to be ineffective [9]. However, that an imbalance in Aβ production and clearance being a universal and critical aspect of AD pathology is a notion that has been extensively validated by animal models and clinical correlations. An important point in this regard is that APP proteolytic processing need not always produce the amyloidogenic Aβ peptides. If APP is first subjected to cleavage by an α-secretase [10], such as a disintegrin and metalloproteinase (ADAM)10 [11,12] or the tumor necrosis factor-alpha converting enzyme (TACE/ADAM17) [13], the BACE1 recognition site is disrupted and the subsequent γ-secretase cleavage does not produce Aβ (see Figure 1). In its place, a fragment known as p3 or Aβ_17–42_ is generated. Although p3 is known to be found in aggregated form in Down’s syndrome patients’ preamyloid lesions [14] and could also be neurotoxic at high concentrations [15], it is generally perceived to be a much milder pathological agent compared to BACE1-produced Aβs. Furthermore, the soluble product sAPPα thus generated is known to be neuroprotective [16,17,18], and particularly in the context of the AD brain, could act against the neurotoxicity of Aβ [19].

The ectodomain cleaving sheddase ADAM10 has a number of important brain substrates such as Notch [20] (and its ligands [21]) and N-cadherin [20,22], as well as many different functions [23]. It is essential for neurodevelopment [24] and its aberrant activity has been linked to several neurological diseases other than AD [25,26]. There is also some evidence for a genetic link between ADAM10 polymorphism and sporadic AD [27,28,29,30]. As a pathophysiologically-relevant APP α-secretase [12], many AD symptom-attenuating effects of experimental manipulations and drugs/compounds have been attributed to an enhancement of α-secretase-based APP processing, some of which are directly linked to ADAM10 activity [31,32,33,34,35]. APP processing in AD is most relevant in neurons and the basal activity of ADAM10 in neurons is influenced by a myriad of regulators at the transcriptional and translational levels, as well as by modulations occurring post-translationally (reviewed in [34,36]). Manipulation of ADAM10 expression and/or activity is thus an often contemplated therapeutic approach to AD. However, ADAM10 activity’s association with inflammation and cancers [37,38,39,40,41,42] is an important caveat that may have stifled interest or progress in this regard. 

Conversely, dysregulation of endogenous ADAM10 inhibitors may contribute to AD pathogenesis. Known endogenous ADAM10 inhibitors include the reversion-inducing cysteine-rich protein with Kazal motifs (RECK) [43] and the secreted-frizzled-related proteins (SFRPs) [44]. A recent finding by Esteve and colleagues that SFRP1 is elevated in AD brains and contributes to AD pathogenesis, is of great importance and uncovers SFRP1 as a novel AD therapeutic target [45]. In the paragraphs below, I shall discuss SFRP1′s role in AD as deciphered by the authors, its potential as a target for AD therapy, and importantly, the reservations associated with the promises. 

## 2. Multiple Ways of Modulating α-secretase-Based APP Processing

A quick survey of experimental manipulations with AD models in which symptomatic or neuropathological improvements have been attributed to an enhanced α-secretase-based APP processing is in order. Transgenic neuronal over-expression of ADAM10 in mice reduced Aβ and plaque formation while increasing sAPPα secretion, which translated into an alleviation of deficits in long term potentiation (LTP) or cognitive impairment [46]. Other more indirect manipulations include changes to membrane trafficking, neural activity, hormones and growth factors, small molecules and activators of ADAM10, as well as the activation of various signaling pathways. A primary determinant of whether APP is first subjected to α- or β-cleavage is dependent on its co-trafficking and membrane residence with BACE1 and ADAM10. In this regard, modulators of exocytic and endocytic membrane traffic such as members of the sorting nexin family SNX27 [47] and SNX8 [48], the polarity protein Par3 [49], the microtubule binding protein superior cervical ganglion 10 (SCG10) and endocytic itinerary regulators such as huntingtin-associated protein 1 (HAP1), have all been shown to promote non-amyloidogenic APP processing. The trafficking and stabilization of ADAM10 and its substrates APP and Notch at the plasma membrane and endosomes are also regulated by members of the tetraspanin family [50,51,52]. Furthermore, acetylcholinesterase inhibitors such as donepezil, which are frontline drugs administered to AD patients, have also been shown to promote ADAM10 trafficking [53].

Both the activation of the excitatory glutamate receptors N-methyl-d-aspartic acid (NMDA) [54] and the α-amino-3-hydroxy-5-methyl-4-isoxazolepropionic acid (AMPA) [55] receptors, promote non-amyloidogenic APP processing, the former via the upregulation of ADAM10 through Wnt/MAP kinase signaling. Serotonin type 4 receptors physically interact with ADAM10 and agonist stimulation in known to increase sAPPα secretion [56]. As Aβ dysregulates AMPA and NMDA receptor trafficking and thus neuronal activity [57,58], amyloidogenesis thus reciprocally inhibits the non-amyloidogenic pathway. A number of hormones and growth factors are also known to enhance α-secretase-based APP processing. Estrogen is known to be neuroprotective against AD [59], and at least one important aspect of this protection is via enhancement of α-secretase processing [60,61]. The beneficial effect of the neuroprotective catechin (-)-epigallocatechin-3-gallate (EGCG) in AD mice has been shown to be exerted through estrogen receptor-mediated activation of ADAM10 [62]. The pineal hormone melatonin’s beneficial effect in AD pathology is multifaceted [63], but stimulation of α-secretase-based processing of APP through upregulation of ADAM10 and ADAM17 would be an important aspect [64]. Retinoic acid, acting through retinoic receptor-α, is known to be neuroprotective in AD via transcriptional upregulation of ADAM10 [65,66,67]. In a placebo-controlled double-blind study, administration of a synthetic retinoid acitretin to 21 patients with mild to moderate AD was well-tolerated and resulted in a significant increase in sAPPα in the cerebrospinal fluid (CSF) [68], which attested to the safety and effectiveness of pharmacologically inducing α-secretase processing in a clinical setting. The vitamin E α-tocopherol [69] and the omega-3 fatty acid docosahexaenoic acid (DHA) could also promote α-secretase-based processing albeit through less understood mechanisms [70]. There is some evidence that the neurotrophins, such as nerve growth factor [71,72] and brain-derived nerve growth factor [73], also promote α-secretase-based APP processing, and this would likely constitute an important aspect of their neuroprotective actions in AD.

Perturbations of certain signaling pathways could also lead to an enhanced α-secretase-based processing of APP [74]. α2-adrenergic agonists reduced Aβ levels while significantly increasing APPα levels in an experimental glaucoma model [75]. Cholesterol levels are known to have a tight association with AD, and hypercholesterolemia is an AD risk factor [76,77]. Inhibition of cholesterol synthesis by statins, inhibitors of the hydroxymethyl glutaryl-CoA (HMG-CoA) reductase, stimulated sAPPα production and ameliorated AD phenotype [78,79,80]. However, the effect of cholesterol lowering is not limited to α-secretase-based APP processing, as very recent studies indicate that cholesterol also regulates tau pathology independently from Aβ [81]. The inhibition of histone deacetylases (HDACs) is also known to be neuroprotective, and Class 1/II HDAC inhibitors increased ADAM10 expression [82,83]. On the other hand, the activity of SIRT1, a NAD^+^-dependent class III histone deacetylase, is beneficial in AD [84]. SIRT1 has a myriad of neuroprotective activities but it is known to upregulate ADAM10 expression through retinoid acid receptors [85,86]. A good number of compounds with varying chemical backbones also enhance non-amyloidogenic processing of APP with mechanisms that have not been clearly deciphered [87,88,89,90,91,92,93,94], and some of which may involve ADAM10 activation. ADAM10’s activity on APP is also known to be dependent on membrane lipids factors, such as transient exposure of the negatively charged phospholipid phosphatidylserine (PS) [95], as well as O-GlcNAcylation of APP [96].

On the other hand, ADAM10 expression is negatively regulated via transcriptional repression by factors such as T-box transcription factor 2 (TBX2) [97], and by a number of micro (mi)RNAs [98,99,100,101]. At the protein or enzymatic level, ADAM10 activity is known to be inhibited by some members of the tissue inhibitor of metalloproteinases (TIMP) family [102,103], which are endogenous inhibitors of matrix metalloproteinases (MMPs) [104]. Likewise, the MMP inhibitor RECK [105] regulates ADAM10 by directly inhibiting its enzyme activity [43]. In the brain, ADAM10 activity is also inhibited by members of the SFRP family [44], which are antagonists of Wnt signaling [106,107]. SFRP1, which is expressed in some brain cell types, turns out to have a potentially important role in AD [45], a finding which we shall now turn our attention to. 

## 3. Secreted-Frizzled-Related Protein 1 and Alzheimer’s Disease

Given ADAM10’s role in AD, one might expect that either ADAM10 levels or activity may be somewhat compromised under disease conditions. Indeed, a recent study has indicated that ADAM10 levels are reduced in the CSF of AD patients compared to control [108]. The underlying reason for this reduction is yet unclear. However, a further conjecture is that independent of its levels, ADAM10 activity may also be somehow reduced in the AD brain. Esteve and colleagues had precisely this hypothesis in mind as they have discovered SFRP1 and 2 as endogenous inhibitor of ADAM10 [44]. Known to be regulators of Wnt signaling [106,107], SFRPs also bind ADAM10 and inhibit the latter’s activity. As a result, Notch signaling was transiently upregulated in *Sfrp1*^−/−^; *Sfrp2*^−/−^ embryos [44].

In a recent report, Esteve and colleagues have asked if SFRP1 levels were altered in AD [45]. Using an antibody raised against SFRP1, the authors found that the protein was elevated in detergent extracts of entorhinal and frontal cortex from AD patients at different stages (presymptomatic, mild and advanced according to the Braak and Braak (BB) scale) compared to age-matched controls. AD patients also had elevated SFRP1 levels in their CSF. Correspondingly, sAPPα levels were significantly lower in extracts from AD patients, indicating that SFRP1 elevation correlated with a reduction in non-amyloidogenic APP processing. The increase in SFRP1 protein level was parallel to that of *SFRP1* transcript, and *SFPR1* has in fact been noted in earlier microarray analyses to be among those genes that are progressive induced in the hippocampus of incipient or advanced AD patients [109]. Immunofluorescence analyses revealed prominent SFRP1 signals in elastin-positive blood vessels colocalizing with Aβ deposits, in some GFAP-positive reactive astrocytes surrounding the amyloid plaques, as well as in activated Iba1-positive microglia infiltrating in the plaques. Interestingly, immunohistochemical analysis also showed specific accumulation of SFRP1 in the core of amyloid plaques, suggesting that SFRP1 may also interact directly with Aβ peptides or their oligomers, and could perhaps co-aggregate with the latter. Indeed, aggregation assays showed that SFRP1 and Aβ could be found in SDS-resistant complexes. Ultrastructural analysis indicated that these two polypeptides interfered with each other’s aggregation norms, at least in vitro. Notably, the presence of SFRP1 appears to disrupt fibril/protofibril formation by Aβ.

Is the SFRP1 elevation causally linked to disease progression in AD? What is seen with human AD brain samples was well recapitulated in an AD transgenic mouse model (harboring mutated APP (APP695swe) and Presinilin1 (PS1-dE9) under the prion promoter (APP; PS1)), and these changes were already evident in pre-symptomatic 2-month-old mice. Furthermore, co-immunoprecipitation analyses of cortical extracts of 6-month-old APP;PS1 mice showed that SFRP1 and Aβ do interact in vivo. APP processing occurs mostly in neurons, and SFRP1 was indeed found in synaptosomal preparations from 8-month-old APP;PS1 mice, where it could be specifically co-immunoprecipitated with ADAM10 [45]. *SFPR1* transcripts were, however, localized mostly to astrocytes, microglial and choroid plexus cells, and SFRP1-positive cells surrounding the amyloid plaques appeared to express more *SFRP1* mRNA than those that were located more remotely. Exogenous expression of SFRP1 in heterozygous APP;PS1 mice (with slowed amyloid plaque build-up compared to the homozygous animal) by lentiviral transduction accelerated amyloid plaque formation, and these were surrounded by an elevated number of CD45-positive activated microglia (which is indicative of gliosis), as well as by lysosomal-marker-enriched dystrophic neurites. On the other hand, APP;PS1 mice rendered SFRP1-null by crossing with *SFRP1^−/−^* mice developed significantly fewer and smaller plagues, with a correspondingly lower degree of gliosis. Furthermore, the levels of β-secretase products like Aβ42 and CTFβ were reduced, while the α-secretase products CTFα and sAPPα were elevated in the brains of SFRP1-null APP;PS1 (APP;PS1;*Sfrp1^−/−^*) mice. That ADAM10 activity is increased in these mice was also indicated by the enhanced processing of another ADAM10 substrate, N-cadherin. With the levels of APP, BACE1 and ADAM10 remaining largely unaffected by the loss of SFRP1, the pathological features of AD in the mouse model thus appears to be directly and significantly influenced by glia-secreted SFRP1′s suppression of ADAM10’s non-amyloidogenic processing. This loss of SFRP1-induced reduction in histopathological phenotype of the APP;PS1;*Sfrp1^−/−^* mice also translates to a significantly improved performance in novel object recognition and Morris water maze behavioral tests compared to APP;PS1 mice (with *Sfrp1^−/−^* mice’s behavioral performance being indistinguishable from wild type mice).

Given that a loss of SFRP1 alleviated AD histopathological features and behavioral deficits, might targeting SFRP1 be beneficial to the disease? The authors provided some proof of principle in this regard using an IgG1 monoclonal antibody with SFRP1-neutralizing activity. Systemic injection of the antibody through the retro-orbital sinus resulted in detectable brain parenchymal infiltration, with particular accumulations around amyloid plaques. Administration of the antibody significantly reduced the cortical Aβ_42_ levels and plaque burden of APP;PS1 mice, as well as the areas of dystrophic neurites. Furthermore, synaptic functions and long term potentiation (LTP) formation as assessed by field excitatory postsynaptic potential (fEPSPs) recordings between hippocampal CA3 and CA1 cells in acute slices were also significantly improved by a prolonged SFRP1 neutralizing treatment. These findings therefore point towards the targeting of SFRP1 as a potentially viable and effective strategy against AD.

## 4. A View of SFRP1 in AD—Mechanisms, Benefits and Risks

The findings of Esteve and colleagues amounts to the identification of a novel pathological regulator of AD in the form of SFRP1 [45]. The possible roles of SFRP1 in AD are summarized in Figure 1. Although whether other members of the SFRP family [106] play similar roles in this regard is yet unclear, the reduction of AD pathology in the APP;PS1 mouse in the absence of SFRP1 alone suggest that it could well be a major pathological factor in the AD brain. With this finding, attempts to screen for *SFRP1* polymorphisms and variants that might predispose individuals to early- or late-onset AD should now be underway. Several aspects of SFRP1′s action in AD as documented by Esteve and colleagues raise important questions that would deserve further attention, and these are discussed below. 

Firstly, how is SFRP1 expression induced in AD? Dysregulation of *SFRP1* gene expression is better known in the context of cancer, and *SFRP1*-promoter hypermethylation and suppression of expression has been associated with several different cancer types [110]. Of particular relevance to the brain are glioma [111] and astrocytoma [112]. Less is known about how *SFRP1* expression is upregulated. However, hydrogen peroxide has been shown to cause *SFRP1* promoter demethylation [113] and SFRP1 has been found to be over-secreted upon cellular senescence caused by DNA damage or oxidative stress [114]. Given the significant role played by oxidative stress in AD and Aβ production [115,116], it could conceivably underlie the upregulation and hypersecretion of SFRP1. Given that cells surrounding amyloid plaques have elevated *SFRP1* transcripts, there is also a possibility that Aβ itself represents a stress signal that induces SFRP1 expression. All these notions would warrant further investigation and analysis.

Secondly, how does SFRP1 interact with Aβ and amyloid plaques and how might this interaction affect Aβ-associated neuropathology? The results of Esteve and colleagues [45] have indicated that SFRP1 promoted amyloid plaque formation, and that SFRP1 perturbs fibril and protofibril formation by Aβ in vitro. Whether SFRP1 affects oligomerization of Aβ peptides, and how the above actually impacts amyloid plaque formation/deposition is unclear. The smaller plaques in APP;PS1;*Sfrp1^−/−^* mice could be due either to a decrease in Aβ load, or SFRP1′s inhibition of conversion of Aβ oligomers to fibrils/protofibrils, or both the above. Soluble Aβ oligomers are known to be more directly neurotoxic that insoluble Aβ aggregates in plaques [4,117]. SFRP1 could therefore potentially enhance soluble Aβ oligomer-mediated neurotoxicity by inhibiting Aβ fibril formation and aggregation independently of its role in promoting Aβ production via inhibition of ADAM10. On the other hand, it has been shown that the amyloid protofibrils are also critically important neurotoxic species in AD [118], as they stimulate microglial activation [119] and directly perturb membrane integrity [120]. All these possibilities therefore require further exploration and clarification. 

The third question concerns SFRP1′s other known role in inhibiting Wnt signaling. Esteve and colleagues have indicated that SFRP1′s observed activity appeared to be independent from Wnt signaling modulation, as the transcript and protein levels of Axin2, a major Wnt/β-catenin inducible gene product, was not affected by the loss of SFRP1 [45]. However, from the perspective of AD as a progressive disease, Wnt signaling modulation may be important in vivo. In this regard, it is notable that aberrant Wnt signaling is a prominent feature in aging and several brain diseases [121,122], including AD [123,124,125,126,127,128,129,130,131,132]. One underlying cause of Wnt-signaling-pathway defects in AD progression is that Wnt signaling represses the transcript levels of BACE1. In N2a cells, over-expression of Wnt agonist reduced Aβ levels and BACE1 expression, while over-expression of SFRP1 resulted in the opposite effect [126]. Loss of Wnt signaling would thus promote amyloidogenic APP processing, Aβ production [127,132], and downstream pathological events [128,129]. Given that it is an important Wnt signaling inhibitor [106], SFRP1′s inhibition or downregulation could therefore alleviate Aβ pathogenicity [125,132] somewhat independently of its inhibition of ADAM10 and non-amyloidogenic APP processing.

Attempts to diminish Aβ pathology, such as targeting BACE1 [133] or the use of anti-amyloid antibodies [134], have been a prime general strategy against AD. However, despite demonstrated efficacy in animal models, neither approaches has recorded a successful clinical trial to date [135,136]. The active pathological role demonstrated for SFRP1 in the AD mouse model and its correlative elevation in human AD brain samples is suggestive of a therapeutic opportunity. This is further corroborated by the experimental alleviation of AD phenotype using an SFRP1 antibody [45]. The notion of targeting of SFRP1 in AD, however raises some concerns, at least in theory. A principal concern parallel that raised by the strategy of ADAM10 activation, namely the potential of oncogenic induction. ADAM10 is the major source of human epidermal growth factor receptor 2 (HER2) ectodomain shedding activity in HER2-overexpressing breast cancer cells [137,138], and is known to promote cancer cell migration [39,41], invasiveness [42] and metastasis [40] in several contexts of cancer progression, as well as in brain glioma [139]. SFRP1 is negatively associated with cancer development and progression, and hypermethylation of *SFRP1* promoter and transcription suppression [110] has been demonstrated for brain cancers of glial origin, such as glioma [140] and astrocytoma [112]. In this regard, SFRP1′s tumor suppression activity likely also has much to do with its inhibition of Wnt signaling. In the context of brain cancers, the inductive role of Wnt signaling in glioblastoma development is well-known [141,142]. Prolonged inhibition of SFRP1 would therefore increase the risk for cancer, particularly in the aging brain in the context of late-onset AD. SFRP1 was also shown to be anti-apoptotic in fibroblasts through regulation of several apoptosis-related genes [143]. Although whether it has a neuronal survival function per se is yet unclear, inhibition of pathologically active ADAM10 has been recently shown to rescue synaptic disruption and cognitive decline in neurons of Huntington’s disease mice [26]. Loss of SFRP1 activity could also impact Wnt signaling and give rise to other systemic problems. For example, decreased SFRP1 expression is associated with cardiomyopathy resulting from increased Wnt activity in aged hearts [144]. In other words, any SFRP1 targeting would need to be precise and ideally localized or restricted to the brain, which would increases the degree of difficulty in terms of therapeutic implementation. 

## 5. Epilogue

Recent work has identified SFRP1 as a novel regulator of AD pathology and as a potential therapeutic target. Given its inhibition of non-amyloidogenic processing of APP, its potential in interfering with Aβ and amyloid toxicity, as well as its potential modulation of Wnt signaling, SFRP1 appears to be a promising candidate for therapeutic targeting. However, SFRP1′s modulation of ADAM10 and Wnt signaling has a flip side, which includes the promotion of cancer and the negation of other beneficial aspects. The feasibility of SFRP1′s full or systemic ablation may therefore be restricted. Given these caveats, a better understanding of the complexity of SFRP1′s actions in the brain and in AD would be required before a useful SFRP1-based therapeutic strategy could be developed. 

## Abbreviations

Aβ	amyloid beta;
ADAM10	a disintegrin and metalloproteinase 17;
AMPA receptor	α-amino-3-hydroxy-5-methyl-4-isoxazolepropionic acid receptor;
APP	amyloid precursor protein;
CSF	cerebrospinal fluid;
DHA	docosahexaenoic acid;
EGCG	catechin (−)-epigallocatechin-3-gallate;
HAP1	huntingtin-associated protein 1;
HMG-CoA reductase	hydroxymethyl glutaryl-CoA reductase;
MMPs	matrix metalloproteinases;
NAD	nicotinamide adenine dinucleotide;
NMDA receptor	N-methyl-d-aspartic acid receptor;
RECK	reversion-inducing cysteine-rich protein with Kazal motifs;
sAPPα	soluble APPalpha;
sAPPβ	soluble APPβ;
SCG10	superior cervical ganglion 10;
SFRP1	secreted-frizzled-related protein 1;
SNX27	sorting nexin 27;
TACE	tumor necrosis factor-alpha converting enzyme;
TBX2	T-box transcription factor 2
